# Preconditioning with Low-Dose Radiation Improves Antitumor Immunity and Survival in DC-Vaccinated Mice

**DOI:** 10.3390/life15091402

**Published:** 2025-09-04

**Authors:** Eric Kwon, Shelby Namen, Colin J. Willoughby, Solomon Kang, Gaurav Pandey, Alexander B. Kim, Carl J. DeSelm

**Affiliations:** 1 Department of Radiation Oncology, Washington University School of Medicine, St. Louis, MO 63110, USA; erickwon@wustl.edu (E.K.); namensl@wustl.edu (S.N.); c.j.willoughby@wustl.edu (C.J.W.); k.solomon@wustl.edu (S.K.); gpandey@wustl.edu (G.P.); abkim@wustl.edu (A.B.K.); 2 Bursky Center for Human Immunology and Immunotherapy, Washington University School of Medicine, St. Louis, MO 63110, USA

**Keywords:** dendritic cell vaccine, preconditioning regimens, low-dose radiation

## Abstract

Preconditioning regimens are essential for the immunologic success of cell therapies like CAR T cells. Nevertheless, their effect on cancer vaccines is underexplored, and preconditioning regimens are generally absent from cancer vaccine clinical trials. To address this knowledge gap, we evaluated the impact of various preconditioning strategies on dendritic cell (DC) vaccine efficacy in a murine tumor model. Mice bearing syngeneic tumors received preconditioning with 2 Gy low-dose radiation therapy (LD RT; whole-body or tumor-only), cyclophosphamide, paclitaxel, LD RT plus cyclophosphamide, or no preconditioning, followed by administration of antigen-loaded DCs. Whether whole-body or tumor-directed, LD RT preconditioning significantly enhanced vaccine-induced antitumor CD8^+^ T cell responses and improved survival compared to DC vaccine alone and all other preconditioning groups. Cyclophosphamide preconditioning reduced vaccine efficacy and negated the benefits of LD RT, while paclitaxel had no significant effect. Notably, whole-body LD RT induced the strongest tumor antigen-specific T cell response. These findings suggest that preconditioning regimens can significantly influence cancer vaccine outcomes, as in CAR T cell therapy. Rational selection of preconditioning agents may either maximize or minimize the therapeutic potential of DC cancer vaccines and should be considered carefully in clinical trials.

## 1. Introduction

Tumor vaccines—including peptide, mRNA, DNA, and dendritic cell (DC)-based platforms—represent a promising frontier in cancer immunotherapy. Tumor antigen vaccine efficacy depends on endogenous DCs in vivo to capture, process, and present the antigen to T cells, thereby initiating an antitumor immune response [1,2]. In contrast, DC vaccines involve the ex vivo generation and maturation of DCs that are directly loaded with tumor antigens prior to administration. This effectively bypasses the requirement for antigen uptake in vivo but requires the ability to culture the appropriate DC population for in vivo efficacy. All tumor vaccine strategies ultimately seek to amplify the body’s natural antitumor immunity by providing T cells with precise instructions for recognizing and eliminating cancer cells [3]. Among DC subtypes, conventional type 1 dendritic cells (cDC1s) are uniquely capable of cross-presenting antigens and are essential for the activation of CD8^+^ cytotoxic T cell responses against tumors in vivo [4].

A critical, yet underappreciated, aspect of adoptive cell therapies, such as CAR T cell therapy, is the use of preconditioning regimens prior to cell infusion. Lymphodepleting agents, most commonly cyclophosphamide, are routinely administered to enhance therapeutic efficacy by creating a favorable immunologic milieu. Key aspects of lymphodepleting agents include depleting regulatory and suppressive immune populations, increasing homeostatic cytokine availability, and facilitating the expansion and persistence of transferred cells [5,6,7]. Additional agents, such as LD RT [8,9] or paclitaxel [10,11], have been shown to modulate the tumor microenvironment, increase antigen presentation, and promote T cell infiltration. The necessity of preconditioning for optimal CAR T cell efficacy is well established, with improvements in objective response rates and progression-free survival [5,6,7]. Nevertheless, the optimal preconditioning regimens for other types of immunotherapies, including cancer vaccines, have not been well characterized.

In contrast to CAR T cell therapy, preconditioning regimens are not routinely incorporated into clinical trials of cancer vaccines, despite the mechanistic rationale and potential for similar immunomodulatory benefits. Early-phase clinical studies of DC and other cancer vaccines have demonstrated the capacity to increase antitumor T cell responses [12,13,14], but have not yet been translated into clear survival benefits. This discrepancy may partly reflect the absence of intentional preconditioning strategies that could potentiate vaccine-induced immunity by overcoming tumor-mediated immune suppression or enhancing T cell priming and expansion.

To begin to address this critical gap, we systematically evaluated the impact of several of the most commonly used preconditioning regimens for CAR T cell therapy on the efficacy of tumor antigen-loaded DC vaccines in a syngeneic murine tumor model. These regimens included cyclophosphamide, LD RT, and paclitaxel. Our objective was to identify clinically translatable preconditioning strategies that can be further validated and incorporated into future cancer vaccine trials to maximize antitumor immunity and improve patient outcomes. To minimize confounding variables associated with different vaccine platforms, such as mRNA, DNA, or peptide antigens, as well as the use of various adjuvants, we utilized ex vivo antigen-loaded cDC1s, which are the critical DC population for generating antitumor T cell responses in vivo [4]. This approach allowed us to directly assess the effects of preconditioning on the antigen-induced antitumor immune response, independent of antigen uptake or adjuvant effects. Using ovalbumin-loaded cDC1s (Ova DCs) and established Kras;Trp53 (KP) Ova cell tumors, we measured the impact of each preconditioning regimen on tumor growth, survival, and the magnitude of antigen-specific CD8^+^ T cell responses. Notably, of the preconditioning regimens we compared, which included paclitaxel, cyclophosphamide, LD RT, or cyclophosphamide with LD RT, LD RT induced the strongest antigen-specific T cell response. Cyclophosphamide preconditioning reduced vaccine efficacy and negated the benefits of LD RT, while paclitaxel alone had no significant effect. These findings highlight that integrating relatively simple preconditioning regimens into the design of cancer vaccine therapy may significantly impact the antitumor T cell response and clinical outcome.

## 2. Materials and Methods

### 2.1. Mouse Tumor Model

The Kras;Trp53 (KP) Ova cell line was engineered and generously donated by Dr. David DeNardo. The cell line is a KP pancreas adenocarcinoma that expresses cytoplasmic ovalbumin. These cells were cultured on collagen-coated plates in DMEM/F-12 Ham (Cat. D6421, Millipore Sigma, Burlington, MA, USA) supplemented with 1% sodium pyruvate (Cat. MT25000CI, Corning) and 1% Penicillin–Streptomycin (Cat. 15140122, Gibco, Grand Island, NY, USA). These cells were given two passages after thawing before injection and not maintained for more than 8 passages before an in vivo experiment. These cells (5 × 10^5^ per 50 uL) were then injected into C57BL/6 mice in the flank subcutaneously. To minimize animal usage in accordance with ethical guidelines and to better model patients with multifocal disease, tumors were injected into both flanks of each mouse. For local irradiation experiments, both tumors received focal irradiation at a dose of 2 Gy.

The mice were shaved for easier access to the subcutaneous layer and measurement of tumor size in the future. The tumors on the mice were measured with a caliper in the longest and shortest diameters in 0.5 mm increments, then averaged to provide an average tumor diameter. These mice were housed under a protocol approved by the Washington University Institutional Animal Care and Use Committee (Prot. 24-0239), according to all relevant animal use guidelines and ethical regulations.

### 2.2. Irradiation

Mice bearing established tumors were randomized on Day 0. On Day 4, preconditioning radiation was delivered using an X-ray irradiator. Animal irradiation was performed using a small animal irradiator (Precision X-Rad i225, 320 kV, 12.5 mA, filter 2 mm Al, SSD 50 cm, collimator 10 cm × 10 cm, single fraction at 150 cGy/min) with open jaws in the anterior–posterior (AP) direction to deliver 2 Gy to the entire animal.

To administer radiation to the tumor only, mice in LD RT groups were anesthetized under isoflurane, and the tumor was treated with a single tangent field. Mice were laid in a prone position with their tails oriented inferiorly. The irradiation field illuminator was used to ensure that the mice were positioned in the correct position. A 5 mm thick lead shield with a cutout for the tumor was placed over the animal to ensure no dose to the total body.

### 2.3. Chemotherapy

Cyclophosphamide (Cat. 13849, Cayman Chemical, Ann Arbor, MI, USA) was resuspended in PBS and injected at 250 mg/kg [15]. Paclitaxel (Cat. T7191, MilliporeSigma, Burlington, MA, USA) was titrated to 20 mg/kg [16] in 20% 50:50 Cremophor EL/ethanol, 80% saline. Both drugs were injected intraperitoneally. Both of these dosages have been chosen to model lymphodepletion in the mice before administration of the DC vaccine, which provides a basis of comparison between these chemotherapies and LD RT as preconditioning regimens.

### 2.4. Dendritic Cell Culture

Femurs and tibias of C57Bl/6 mice were isolated, then a 12,000 rcf × 10 s centrifugation was used to isolate the bone marrow, and RBC lysis (Cat. A10492-01, Gibco, Grand Island, NY, USA) was performed. The cells were cultured as previously described [17] with slight modifications. Briefly, the isolated bone marrow cells were plated into a 10 cm plate in 1.5 × 10^6^ cells/mL using IMDM with HEPES and L-glutamine (Cat. 12440061, Gibco) supplemented with 10% heat-inactivated fetal bovine serum (Cat. 10082147, Gibco), 0.1% 2-mercaptoethanol (BME) (Cat. 21985-023, Gibco), 1% Penicillin–Streptomycin (Cat. 15140122, Gibco), 1% sodium pyruvate (Cat. MT25000CI, Corning, Corning, NY, USA), 1% non-essential amino acids (Cat. MT25025CI, Corning), 2.5 ng/mL rec mouse GM-CSF (Cat. 315-03, Peprotech, Cranbury, NJ, USA), and 5% Flt3L conditioned media [17]. On day 5, additional complete IMDM media with BME was added to the culture on top of the original media. On day 9, a full media change was conducted with fresh IMDM media, including GM-CSF and 5% Flt3L-Fc conditioned media, and cells were expanded into 0.3 × 10^6^ cells/mL into a T175 flask.

**Figure 1:**
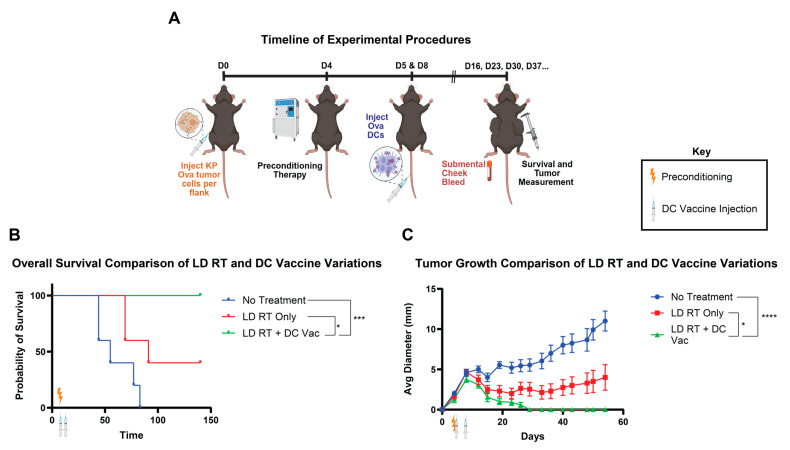
Dendritic cell vaccine with LD RT significantly improves survival over LD RT alone. (**A**) Mice were injected with ova-expressing KP pancreas tumor cells into the flank on day 0. Preconditioning therapy of 2 Gy RT to the whole body was given on day 4. Bone marrow Flt3L-differentiated DCs were electroporated with the ovalbumin protein (Ova DCs) and injected into tumor-bearing mice through the tail vein on days 5 (1 × 10^6^) and 8 (1 × 10^6^). (**B**) Combined survival curves from the indicated groups in this experiment. For comparison of survival curves, the log-rank (Mantel–Cox) test was performed. *p* ≤ 0.05 (*), *p* ≤ 0.001 (***). (**C**) Tumor diameter and mouse survival rates were recorded over time. Data are presented as mean ± SEM. *p* ≤ 0.05 (*), *p* ≤ 0.001 (***), *p* ≤ 0.0001 (****). Values from individual days were compared using multiple unpaired *t*-tests with the two-stage step-up method of Benjamini, Krieger, and Yekutieli to correct for FDR and multiple comparisons. If tumor diameter was significantly different between groups on two or more occasions, significance was assigned.

On day 16, cells were analyzed for the proportion of cDC1s in the culture. On day 17, the cells were collected from the culture and electroporated in an Invitrogen Neon NxT with ovalbumin protein. After letting the cells rest for 2–3 h, flow cytometry was performed again on the cells using the abovementioned stain to determine the cDC1 counts after the electroporation process. Using these numbers, the cells were resuspended into PBS and were then injected into the mouse tumor models through tail vein injections (i.v.). To exploit radiation-induced antigen release and inflammatory signaling, DC vaccination was scheduled after a 24-h lag. Antigen-loaded cDC1s were administered at 24 h post-RT (Day 5). Booster vaccinations were given on Day 8 to reinforce clonal expansion and promote memory T cells. A 24-h post-RT interval was selected to coincide with peak availability of tumor antigens and upregulated chemokines that favor DC activation and T cell priming. A boosting dose in a 3-day interval aligned with the expansion-to-contraction transition of the primary response, aiming to enhance magnitude and durability (Figure 1A).

### 2.5. Submental Vein Blood Collection

Submental blood collection was conducted on day 16 and every 7 days after. If the submental vein was not accessible or did not yield enough blood, then the submandibular vein was used. Five drops, or around 250 μL, were collected from each mouse and gently mixed in EDTA tubes. Red blood cells were then lysed with RBC lysis buffer (Cat. A10492-01, Gibco), and remaining cells were stained with two SIINFEKL tetramer stains and T cell markers. Flow cytometry was then performed to analyze SIINFEKL-specific CD8^+^ T cells.

### 2.6. Flow Cytometry

All antibodies were titrated. Fc Receptor Binding Inhibitor Antibody Human (Cat. 14-9161-73, eBioscience, San Diego, CA, USA) was used to block Fc receptors. All flow cytometry was conducted on a Northern Lights 3000 Flow Cytometer (Cytek, Fremont, CA, USA). All flow cytometry analysis was performed on FlowJo v10.9.

DCs on day 16 were stained with XCR1 Brilliant Violet 650 (Cat. 148220, BioLegend, San Diego, CA, USA), MHCII violetFluor 450 (Cat. 20-5321-U100, BD Biosciences, Franklin Lakes, NJ, USA), B220 Brilliant Violet 510 (Cat. 583103, BioLegend), SIRPα PerCP-eFluor 710 (Cat. 46-1721-82, Invitrogen, Carlsbad, CA, USA), and CD11c Brilliant Violet 785 (Cat. 117335, BioLegend).

T cells from submental vein blood were stained with two SIINFEKL tetramers, one conjugated with PE and the other with APC. After the tetramer stains, the cells were stained with CD3 Brilliant Violet 480 (Cat. 565642, BD Biosciences), CD4 PE cy7 (Cat. BDB563933, Biolegend), CD8 Brilliant Violet 570 (Cat. 100739, BioLegend), TCR-β Alexa Fluor 700 (Cat. 109224, BioLegend), CD45 PE Cy5 (Cat. 103109, BioLegend), CD44 APC Vio 770 (Cat. 130124708, Miltenyl Biotec, Bergisch Gladbach, Germany), and CD62L Brilliant Violet 510 (Cat. 104441, BioLegend).

### 2.7. Statistics and Data

All experimental data are presented as mean ± s.e.m. No statistical methods were used to predetermine sample size. Survival analyses were performed using Kaplan–Meier log-rank. Statistical analysis was performed on GraphPad Prism 10 software. Schematic figures were made with BioRender 201 (BioRender.com). All in vivo studies were performed using 5 mice per group.

## 3. Results

### 3.1. DC Vaccine Added to LD RT Significantly Improves Efficacy over LD RT Alone

LD RT has proven to be effective in animal studies as a preconditioning regimen for cellular therapies [8,9]. We first tested whether adding LD RT preconditioning before DC tumor vaccine improved tumor rejection using a tumor model that is not rejected with tumor vaccine alone. The preconditioning regimen was tested on Kras;Trp53 (KP) pancreatic tumor cells expressing ovalbumin (KP Ova), injected into the flank. KP Ova, despite expressing the ova model antigen, which is capable of being recognized by T cells, is an “immune cold” tumor that is unresponsive to anti-PD-1 plus anti-CTLA4 immune checkpoint inhibition [18]. A radiation dose of 2 Gy was used for LD RT, since this dose is below definitive clinical doses for solid tumors and is well tolerated but still has cellular and immunomodulatory effects [8,9]. LD RT was administered to the mouse four days after subcutaneous tumor injection. Subsequently, Ova DCs were injected intravenously (IV) on days 5 and 8 (Figure 1A). LD RT alone led to growth reduction but did not eliminate tumors. When LD RT was followed by DC vaccine, all tumors were rejected, indicating that the addition of DC vaccine to LD RT improves tumor rejection and survival compared to LD RT alone (Figure 1B,C). Having established that the combination of LD RT with DC vaccine is more effective than LD RT alone, we next asked the reciprocal question—whether the combination of LD RT with DC vaccine is more effective than vaccine alone. Further, we asked whether an optimal preconditioning regimen exists among LD RT and two promising chemotherapy agents (cyclophosphamide and paclitaxel) that are also known to modulate the tumor microenvironment.

### 3.2. LD RT Added to DC Vaccine Significantly Improves Efficacy over DC Vaccine Alone

Since LD RT with DC vaccine led to 100% rejection in our prior experiment, we reduced DC numbers to attempt to achieve more partial responses and allow for survival distinction between groups. Cyclophosphamide is a standard preconditioning agent for CAR T cell immunotherapies [6]; however, a major goal of preconditioning for CAR T cells is to lymphodeplete, making physical space for injected CAR T cells and leading to the release of T cell-supporting cytokines, which help newly injected CAR T cells expand and survive. We used a cyclophosphamide dosage of 250 mg/kg, a standard regimen for other cellular immunotherapies [19]. LD RT and cyclophosphamide were used in one mouse cohort to test whether these therapies work more effectively together, a strategy that is also tested in clinical trials for CAR T cells (NCT06623630). Paclitaxel was utilized in a separate cohort at 20 mg/kg, which has shown promising preconditioning effects in mouse models [11]. All of these preconditioning regimens were applied to mice with established tumors, and Ova DCs were given intravenously on days 5 and 8 at a lower “stress test” dose than in the first experimental design (1 × 10^6^ and 1.5 × 10^5^, versus 1 × 10^6^ and 1 × 10^6^) to better discern potentially subtle differences between each preconditioning regimen.

**Figure 2:**
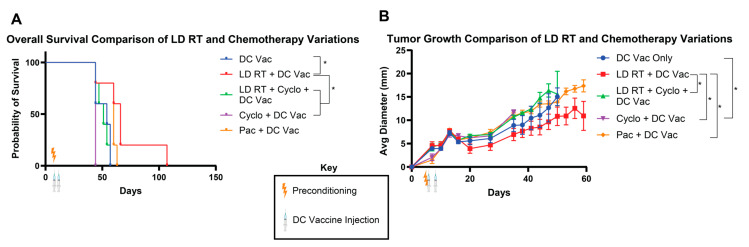
LD RT preconditioning before DC vaccine improves survival over DC vaccine alone, and cyclophosphamide abrogates the benefit of LD RT. As in Figure 1A, KP Ova cells were injected in C57BL/6 mice in the flank subcutaneously. Four days later, mice were irradiated with 2 Gy (LD RT) and/or injected IP with cyclophosphamide (250 mg/kg) or paclitaxel (20 mg/kg). Ova-loaded DCs were injected in the tail vein intravenously on day 5 (1 × 10^6^) and day 8 (1.5 × 10^5^). (**A**) Mouse survival was recorded. Log-rank (Mantel–Cox) tests were conducted to detect significant survival differences. (**B**) Tumor growth was monitored over time. Data are presented as mean ± SEM. *p* ≤ 0.05 (*). Values from individual days were compared using multiple unpaired *t*-tests with the two-stage step-up method of Benjamini, Krieger, and Yekutieli to correct for FDR and multiple comparisons. If tumor diameter was significantly different between groups on two or more occasions, significance was assigned for the group.

Although numbers of mice and clinical differences were relatively modest, the greatest benefit on long-term survival (surviving to day 107) and tumor growth was provided by the LD RT + DC vac group, which improved survival over groups treated with LD RT-cyclophosphamide + DC vaccine, cyclophosphamide + DC vaccine, and DC vaccine only (Figure 2A). Paclitaxel preconditioning led to significantly slower tumor growth but non-significantly improved survival over DC vaccine alone (63 days vs. 57 days, respectively) (Figure 2A,B). Cyclophosphamide preconditioning led to the worst overall survival and the fastest rate of tumor growth (Figure 2A,B); adding cyclophosphamide prior to DC tumor vaccine led to significantly faster tumor growth than DC tumor vaccine alone (Figure 2B). Additionally, adding cyclophosphamide to LD RT abrogated the benefit of LD RT (Figure 2A,B).

### 3.3. Whole-Body LD RT and Tumor-Directed LD RT Significantly Improve DC Vaccine Efficacy

**Figure 3:**
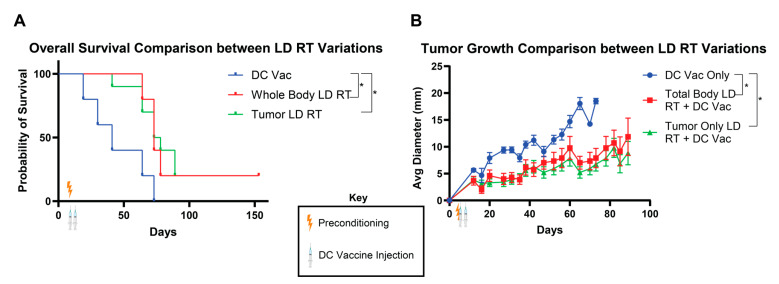
Total-body and tumor-directed LD RT similarly improve the efficacy of DC vaccination. As in Figure 1A, KP Ova cells were injected into C57BL/6 mice in the flank subcutaneously on day 0. Then, the mice were irradiated with 2 Gy across the whole body or to the tumor only on day 4. Ova DCs were injected on day 5 (1 × 10^6^) and day 8 (1.0 × 10^5^). (**A**) Combined survival curves. For comparison of survival curves, the log-rank (Mantel–Cox) test was applied. *p* ≤ 0.05 (*). (**B**) Tumor diameter recorded over time. Data are presented as mean ± SEM. *p* ≤ 0.05 (*). Values from individual days were compared using multiple unpaired *t*-tests with the two-stage step-up method of Benjamini, Krieger, and Yekutieli to correct for FDR and multiple comparisons. If tumor diameter was significantly different between groups on two or more occasions, significance was assigned.

LD RT preconditioning with the DC vaccine produced the greatest overall survival of the preconditioning regimens we tested. LD RT was administered to the entire body in these studies for simplicity, but clinically, patients are treated with targeted radiation to sites of known disease. There may be divergent outcomes of total-body LD RT versus tumor-directed LD RT for DC vaccine preconditioning. To investigate potential differences between these two radiation modalities, we compared the effect of LD RT given to the whole body versus the tumor only prior to DC vaccine treatment. We found that tumor-bearing mice treated with either total-body or tumor-only LD RT significantly improved survival over DC vaccine alone (Figure 3A), and both radiation preconditioning approaches exhibited similarly reduced tumor growth rates (Figure 3B). Compared to the DC vaccine alone, whose longest survival was recorded at 62 days, the total-body LD RT group included mice that survived throughout this study, surpassing 160 days. Survival outcomes for both radiation modalities were comparable and without statistically significant differences.

### 3.4. Whole-Body LD RT Results in Significant Increases in Tumor-Specific CD8^+^ T Cell Populations

**Figure 4:**
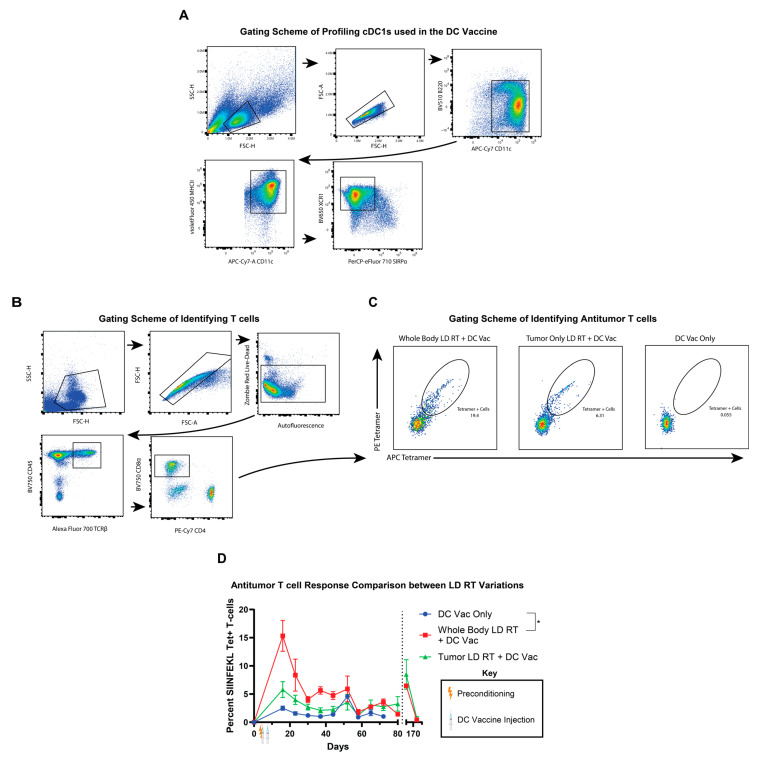
Total-body LD RT improves TCR-specific responses to DC vaccination. (**A**) Representative flow cytometry plots of bone marrow-derived cDC1s (DCs) used in these mouse experiments. Sequential gating includes B220lo CD11c^+^ MHC II^+^ XCR1^+^ SIRPα cells. (**B**) Submental cheek bleed samples were stained for T cell receptors (TCR) specific for MHCI:SIINFEKL (Ova peptide) tetramer complexes, revealing tumor-specific T cells. Representative gating strategy for identifying tetramer-positive CD8⁺ T cells from submandibular blood using flow cytometry is shown with sequential gating, including live cells CD45^+^ TCRb^+^ CD8^+^ tetramer^+^ T cells. (**C**) Representative flow plots of tetramer (SIINFEKL)-specific T cells from whole-body LD RT + DC vaccine treatment on the left, tumor-only LD RT + DC vaccine treatment in the middle, and DC vaccine only treatment on the right. (**D**) Percentages of tetramer-positive CD8⁺ T cells of all CD8^+^ T cells from submental venous blood are shown with SEM over time. Mice treated with the indicated therapy (DC vaccine only in blue circles, whole body LD RT followed by DC vaccine in red squares, or tumor-targeted LD RT followed by DC vaccine in green triangles) that obtained complete rejection and survived >100 days were rechallenged on day 159 with a KP Ova tumor with an injection of 5 × 10^5^ cells into the flank, and tetramer^+^ T cells in the blood were monitored thereafter. Values from individual days were compared using multiple unpaired *t*-tests with the two-stage step-up method of Benjamini, Krieger, and Yekutieli to correct for FDR and multiple comparisons. If values were significantly different between groups on two or more dates, significance for the group was assigned. Data are presented as mean ± SEM. *p* ≤ 0.05 (*).

Since RT can directly affect or kill T cells, even at low doses, we aimed to define the tumor-specific CD8^+^ T cell response after either total-body or tumor-only LD RT preconditioning combined with DC vaccine therapy, compared with DC vaccine therapy alone, to try to understand further the potential mechanism by which LD RT improves tumor growth and mouse survival. Submental cheek bleeds were performed weekly to analyze the SIINFEKL-specific CD8⁺ T cells through tetramer staining (Figure 4A,B). Whole-body LD RT elicited the highest percentage of SIINFEKL-specific CD8^+^ T cells amongst all CD8^+^ T cells (Figure 4C,D). Tumor-only LD RT, prior to DC vaccine, was eliciting an intermediate tetramer^+^ T cell response. While there were no long-term survivors after the DC vaccine alone, there were long-term survivors after tumor-only and whole-body LD RT preconditioning plus DC vaccine conditions, and we re-challenged these mice to assess memory T cell responses. After re-injecting these surviving mice with tumor, a re-emergence of SIINFEKL-specific CD8^+^ T cells was seen in both the tumor-only and whole-body LD RT preconditioning groups. Further, re-injected tumors were eliminated without a visible tumor reappearing. These data demonstrate that LD RT of 2 Gy with DC vaccine generates a stronger antitumor T cell response than DC vaccine alone and that these antitumor T cells are long-lived and capable of responding to tumor re-challenge. In addition, the total-body RT of 2 Gy does not substantially impair or improve the antitumor T cell response compared with local tumor-directed RT, despite the T cells themselves also being subjected to 2 Gy RT (Figure 4D).

## 4. Discussion

In this study, we investigated whether preconditioning with cyclophosphamide, paclitaxel, LD RT, or LD RT plus cyclophosphamide could enhance DC vaccination against KP Ova tumors and found that LD RT preconditioning significantly improved survival compared to DC vaccine alone and all other preconditioning groups.

Paclitaxel had no significant effect, and cyclophosphamide preconditioning reduced vaccine efficacy and negated the benefits of LD RT. We also compared targeted versus total-body LD RT, as the latter may impact immune progenitors. LD RT, whether local or systemic, was more effective at slowing tumor growth and extending survival than DC vaccine alone or with cyclophosphamide or paclitaxel. These results align with prior studies showing LD RT benefits in DC therapy, likely through increased MHC I expression on tumor cells [20] and transient lymphodepletion and homeostatic cytokine release [21,22], which together enhance T cell responses.

Cyclophosphamide and paclitaxel did not improve outcomes over DC vaccine alone, though optimal dosing was not explored. Notably, adding cyclophosphamide to LD RT negated the benefit of LD RT, and using cyclophosphamide before the DC vaccine was the only condition that led to faster tumor growth and shorter survival than the vaccine alone, suggesting that while cyclophosphamide is useful in CAR T cell regimens, it may be detrimental for vaccines relying on endogenous T cells due to its strong lymphodepleting effects.

Both whole-body and tumor-directed LD RT significantly delayed tumor progression and improved survival compared to no preconditioning. Local and systemic LD RT were similarly effective for tumor control, indicating that local modulation may suffice for patients with localized or oligometastatic disease. Systemic (whole-body) LD RT, however, induced higher frequencies of circulating antigen-specific (tetramer-positive) CD8⁺ T cells, though this difference did not translate into better tumor control or survival. Although T cells are very sensitive to radiation, these data reassuringly suggest that whole-body irradiation of 2 Gy does not systemically deplete or damage antitumor T cells enough to hamper the effect of a DC vaccine and instead actually improves the antitumor T cell response, despite all the antitumor T cells also having received 2 Gy RT.

In radiobiology, “low-dose radiation” (LDR) is often defined mechanistically as exposures ≤0.1–0.5 Gy, particularly in studies investigating DNA damage responses. Our study, however, is focused on clinical translation, where the relevant definition derives from patient care rather than cellular thresholds. In clinical practice, some of the lowest radiation regimens include 2 Gy × 2 for palliation in lymphoma, 4 Gy × 5 for palliation, and 6–8 Gy × 1 in frail patients for symptom control (NCCN, EORTC, and ASTRO Clinical Practice Guidelines). Within this context, a single 2 Gy fraction represents the minimal conventional dose that is routinely feasible in patients. We therefore adopt the translational nomenclature used in other clinical and pre-clinical studies [23] and refer to 2 Gy as “low-dose” in this manuscript. We further chose this dose, as it is low enough to be extremely safe but high enough to have known cellular and immunomodulatory effects [8,9].

Mice cured with LD RT and DC vaccine rejected tumor rechallenge and showed renewed expansion of antigen-specific CD8⁺ T cells, indicating durable memory. This demonstrates that LD RT preconditioning with DC vaccine is an effective strategy for short-term responses as well as long-term surveillance. Previous findings also demonstrate that radiation promotes memory T cell formation through inflammatory mediators like type I interferons and IL-12, which aid in memory precursor differentiation and persistence [24,25].

We used Ova DCs as a controlled model to assess how preconditioning shapes T cell responses. This approach allows precise evaluation of preconditioning effects and is likely relevant to other antigen delivery strategies, such as DNA, mRNA, or peptide vaccines, which also depend on DC-mediated T cell priming. Thus, our findings may inform optimization of a broad range of vaccine platforms.

These studies have limitations. Each in vivo experiment was performed once with *n* = 5 mice per group, with a single tumor model. For broader generalizations, these experiments should be replicated in other models, potentially including an immune “hot” tumor, and ideally also in an orthotopic tumor. Whether the statistically significant differences in tumor growth will be clinically meaningful remains to be seen with larger numbers and, ultimately, after human translation.

The mechanistic understanding of the impact of LD RT on DC vaccination is incomplete, and further research is also needed to define the underlying mechanisms producing the results shown. Likely, many mechanisms are at play. It has been shown that sub-lethal irradiation (≤2 Gy) increases β-2-microglobulin and MHC-I expression, proteasome activity, and ultimately MHC-I peptide display on tumor cells [26]. Furthermore, 0.5–2 Gy fractions skew the intratumoral T-cell repertoire toward effector memory phenotypes while sparing overall lymphocyte numbers [27]. A single 0.5 Gy dose is capable of normalizing aberrant tumor vasculature and repolarizing macrophages to an iNOS⁺ M1 state that facilitates CD8⁺ infiltration [28]. Taken together, these studies suggest a unifying model in which low-dose or moderately fractionated radiation transiently “reprograms” the tumor microenvironment without the lymphodepletion that accompanies higher cumulative doses: endothelial normalization and reduced interstitial pressure [28] improve leukocyte trafficking; macrophage repolarization and preserved effector T-cell pools [27] create a more inflammatory cytokine milieu; enhanced antigen-processing machinery on tumor cells [26] increases their visibility to cytotoxic lymphocytes; and sustained, but not excessive, cytosolic-DNA/STING signaling [29] provides the type I interferon burst needed to license dendritic cells. This multifaceted remodeling explains why a single 2 Gy priming dose in our study may convert an otherwise immunosuppressive tumor bed into one that supports robust dendritic cell vaccine-induced T-cell expansion and function.

Radiotherapy can also increase PD-L1 expression in tumors and immune infiltrates [30,31] in a dose-dependent manner [32]. Consistent with this, Azad et al. [33] showed in pancreatic ductal adenocarcinoma models that RT plus gemcitabine upregulated PD-L1 in a JAK/STAT1-dependent manner and that high but not low RT doses synergized with PD-L1 blockade to improve tumor control and augment CD8⁺ T cell infiltration. These findings suggest that checkpoint inhibition may counteract PD-L1–mediated adaptive resistance after RT. Although not included in our current design, evaluating anti-PD-1/PD-L1 therapy in combination with LDR preconditioning and DC vaccination is an important future direction. Such combinations could both amplify priming of tumor-specific T cells and prevent checkpoint-mediated suppression, aligning a vaccine-based approach with current clinical immunotherapy standards.

Conventional 2 Gy/fraction schedules remain the standard globally, yet many preclinical studies underscore that fractionation strongly alters immunologic outcomes [34,35]. Hypofractionated regimens (≥8 Gy) often promote immunogenic cell death, STING-mediated type I interferon production, and synergy with checkpoint blockade, while very low doses (<1 Gy) may remodel the tumor vasculature and macrophage phenotype to allow immune infiltration [36]. By contrast, repeated 2 Gy fractions can induce MHC upregulation but are also associated with chronic lymphopenia and expansion of regulatory populations [34,35]. These findings suggest that in the era of immunotherapy, future protocols may diverge from the universal 2 Gy standard toward tailored fractionation schemes optimized not only for tumor control but also for immune modulation. Within this evolving landscape, our data demonstrate that a single 2 Gy fraction can provide a clinically feasible and immunologically favorable preconditioning for DC vaccination, but other fractionation schemes may be even more beneficial.

**Figure 5:**
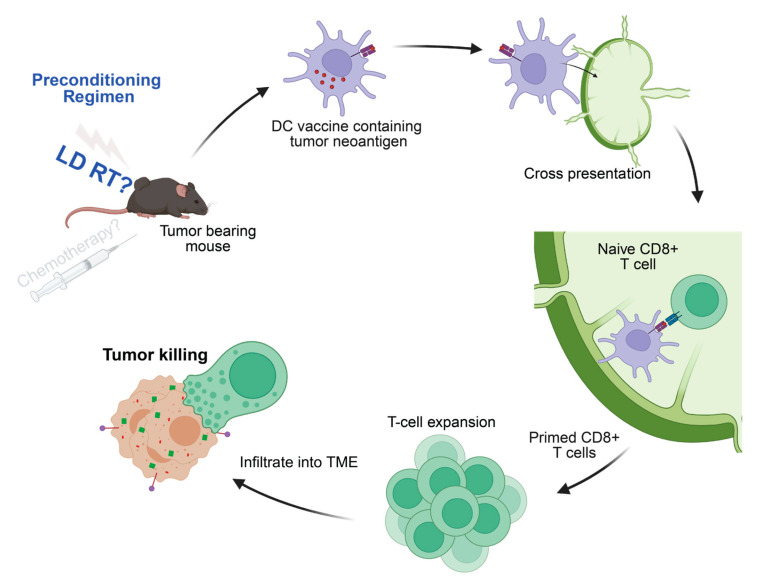
cDC1-derived DC vaccines generate tumor-specific CD8^+^ T cells. Mechanistically, the antigen-loaded DCs travel to lymphatic organs and present the processed tumor antigen (in this case, SIINFEKL peptide) on MHC-I molecules to prime naive antigen-reactive CD8 T cells. Those antitumor CD8 T cells then traffic to tumor sites and perform targeted killing on the antigen (in this case, ovalbumin)-expressing tumors. Our studies demonstrate that LD RT increases the antitumor response created by the DC vaccine.

Our findings resonate with some aspects of previous work on vaccine preconditioning, yet also introduce new, differing observations. Wang et al. [37] demonstrated that ultralow radiation exposure (0.2 Gy) during DC preparation enhanced DC migration, T cell activation, and vaccine efficacy, complementing our finding that clinically relevant 2 Gy preconditioning in vivo improves antigen-specific responses and long-term survival. Similarly, Machiels et al. [38] observed that sequencing immune-modulating chemotherapy with a GM-CSF–secreting whole-cell vaccine augmented antitumor immunity in neu transgenic mice, consistent with the concept that preconditioning can enhance vaccine efficacy. However, in our study, paclitaxel produced modest growth delay without survival benefit, and high-dose cyclophosphamide impaired rather than enhanced vaccine efficacy. These results are consistent with Ghiringhelli et al. [39], who showed that the immunologic impact of cyclophosphamide is strongly dose-dependent: low or “metronomic” regimens selectively deplete regulatory T cells, whereas higher doses can blunt effector function. Together, these studies highlight that preconditioning can strongly influence vaccine outcome (Figure 5), but that the directionality of the effect is dependent on regimen design, underscoring the need for careful translational selection.

## 5. Conclusions

Overall, our results suggest that localized LD RT is sufficient to render the tumor or tumor microenvironment more amenable to antitumor T cell responses induced by DC vaccination, while whole-body LD RT may further amplify primed T cell expansion systemically. Cyclophosphamide preconditioning may impair antitumor T cell response and therefore should be used with caution. These insights can guide further studies to inform clinical strategies that maximize the efficacy of cancer vaccines.

## Data Availability

The data presented in this study are available on request from the corresponding author.

## Abbreviations

The following abbreviations are used in this manuscript:

LD RT	Low-dose radiation therapy
DC	Dendritic cell
cDC1	Conventional type 1 dendritic cell
Ova DCs	Ovalbumin-loaded cDC1s
KP	Kras;Trp53
Flt3L	Fms-like tyrosine kinase 3 ligand
Gy	Gray (unit of radiation dose)
MHC	Major Histocompatibility Complex
SEM	Standard Error of the Mean
TCR	T cell receptor
SIINFEKL	Peptide epitope from ovalbumin that is presented on MHC-I
